# The resilience of the Brazilian Unified Health System is not (only) in responding to disasters

**DOI:** 10.11606/s1518-8787.2024058005731

**Published:** 2024-06-06

**Authors:** Alessandro Jatobá, Paulo Victor Rodrigues de Carvalho

**Affiliations:** I Centro de Estudos Estratégicos Antônio Ivo de Carvalho Fundação Oswaldo Cruz Rio de Janeiro RJ Brasil Fundação Oswaldo Cruz. Centro de Estudos Estratégicos Antônio Ivo de Carvalho. Rio de Janeiro, RJ, Brasil

**Keywords:** Health Policy, Health Systems, Health Management

## Abstract

Coping with the recent COVID-19 pandemic has shown that the Brazilian Unified Health System (SUS) needs to improve its resilience to handle the rapid spread of communicable diseases while ensuring the necessary care for an aging population with comorbidities and in a vulnerable situation. This article identifies, analyzes, and discusses critical aspects of the resilience of the SUS, calling into question the prevailing focus on the robustness and volume of resources mobilized during the outbreak of major disasters. Recent studies demonstrate that the skills that favor adaptation to unexpected situations emerge from the daily functioning of organizations. Restricting the discussion to the mobilization of structures to respond to adverse events has the effect of limiting their potential, inhibiting the emergence of the transformative, adaptive, anticipatory, and learning skills necessary for the sustainable development of resilience.

## INTRODUCTION

Usually, when we discuss the subject of resilience of healthcare organizations, we immediately think of the components that these institutions mobilize to implement the phases of the health disasters response cycle, involving preparation, mitigation, response, and recovery^1^. One of the reasons for this association is because, for a long time, healthcare organizations believed that maintaining or developing physical, financial, and structural resources was sufficient to strengthen their resilience to face the occurrence of emergent health events. After all, according to current definitions, health disasters are intense, unexpected, sudden, and unusual events^2^.

Indeed, this notion of resilience focusing on disaster response is imperative. However, as the bitter experience caused by the COVID-19 pandemic demonstrated, these precautions are insufficient. This became evident when the health systems of the richest countries, considered sound and safe, were strongly impacted by the rapid spread and destructive capacity of the SARS-CoV-2 virus^3,4^.

It took a global health tragedy to establish a new conviction: robust structures are necessary, but not enough to ensure the development of resilience for a nationwide organization like the Brazilian Unified Health System (SUS). In addition to instruments for dealing with disasters, the most recent literature points to the importance of continuous performance in the daily routine of health systems, in normal situations.

Haldane and Morgan^5^ argue that the hard lessons learned during the pandemic represent an opportunity to overcome long-standing problems and structural inequalities in the health sector, in order to optimize the functioning of systems and make the environment more sustainable. Haldane et al.^6^ examined practices in Primary Health Care (PHC) in several countries, including Brazil, indicating that resilience in confronting COVID-19 depended heavily on the extent to which such practices were disseminated and developed, in addition to the structures of services and the volume of available physical and financial resources. Arcuri et al.^7^ highlighted the possibilities for adapting teams from the *Serviço de Atendimento Móvel de Urgência* (Samu-192 – Mobile Emergency Care Service) in the Alto-Solimões (AM) region in the face of increased demand and risks in receiving COVID-19 patients, anticipating adaptive capabilities and weaknesses arising from the operation of the ambulance boat service (popularly called *ambulanchas*), to guarantee access to health care for riverside populations.

With regard to systems governance, recent literature shows how the inadequate functioning of organizational arrangements jeopardized resilience during the pandemic, even with the mobilization of robust structures and extraordinary resources. In this sense, Neves et al.^8^ explored the articulations of interests involved in tackling the pandemic in Brazil, indicating that the incoherence of discourse and disarticulation in official communications negatively interfered with the mobilization of efforts to tackle the spread of the disease. Carvalho et al.^9^ showed that the political agenda at different levels and sectors of government has undermined the system's capabilities to monitor, respond, anticipate, and learn, which are essential aspects of resilient performance. Chioro et al.^10^ go further, stating that the resilience of the SUS fundamentally depends on democratic regimes, in which the basic principles of universality, integrality, and equity would be respected.

The recent recognition by health authorities of the importance of resilience as a fundamental attribute to be developed and managed in national health systems is a positive sign of progress. However, we still need to move towards a conceptualization that broadly considers the functioning of health systems and the articulation between levels of care in favor of the continuous development of preventive, absorptive, and adaptive capabilities, thus incorporating additional possibilities for dealing with the different types of threats to which public health organizations are subject on a daily basis.

## A CONCEPTUALIZATION ADHERENT TO THE SUS

First, it is necessary to highlight that, in fact, the resilience of complex systems involves being able to respond to shocks or disasters. However, in the case of public health, the notion of shock is not limited to a large-scale disruptive event. Unexpected events occur all the time, and even minor events can have a strong destructive potential. Widely known and disseminated practices are subject to the sudden need for adaptations, given the complexity of delivering services integrally and universally, especially to the diverse and unequal Brazilian society.

The resilience of public and universal health systems depends on the way their organizations function and behave in the face of routine adversities faced by the populations they must assist. Therefore, the capabilities that guarantee the security and robustness of systems must be properly managed in favor of resilience, including the uninterrupted, resolutive and quality operation of a minimum set of essential functions^11,12^. This was probably one of the problems that hampered the response to the pandemic in countries like the United States and Brazil. Although they had robust structures, they had to deal with operational and coordination difficulties at the various levels of their health systems.

Brazil faced significant problems in communication and monitoring strategies, the adoption of non-pharmacological measures, the acquisition of vaccines, etc. These issues went beyond the mobilization of physical and financial resources for the SUS and were directly related to the way the system operated during the pandemic, from its governance arrangements to frontline service delivery. With the weakening of social distancing measures, saturation in service capacity led to the suspension of elective surgeries and treatments for chronic diseases in some locations, for example.

Although they have demonstrated the potential for resilience by providing resources to deal with some of the consequences of the pandemic, the SUS and other systems considered safe and robust showed weaknesses that should not be overlooked.

According to Hollnagel^13^, it is not about a system *being* resilient or *having* resilience, but rather *functioning* in a more or less resilient way, which involves being successful under variable conditions. More precisely, resilient performance can be understood as a dynamic and continuous condition, in which problems are momentarily under control due to compensatory changes provided by the players involved. This is essential for the SUS, which has the mission of providing universal, equitable access and comprehensive care in a context in which unexpected changes can arise at any time, with consequences capable of spreading quickly.

Health authorities must develop a culture of permanent transformation that fosters the continuous improvement of preventive, absorptive, and adaptive skills, so that both organizations and people learn from experience, monitor and anticipate risks, and thus respond appropriately to both minor and major public health events. Their structures need to be mobilized and articulated so that resilient performance can always emerge in the face of an unexpected event, regardless of its intensity or destructive potential.

## SCENARIO AND PERSPECTIVES FOR RESEARCH AND PUBLIC POLICY

As part of efforts to tackle the consequences of climate change, the World Health Organization (WHO) has proposed a conceptual framework to promote the development of adaptive capabilities in health systems within the Sustainable Development Goals (SDGs) of the 2030 Agenda^14,15^. According to the WHO, resilience can be strengthened by a set of skills, such as perception, transformation, mobilization, self-regulation, integration, and diversity, all of which are focused on sustaining a minimum level of continuous operation of essential functions for the health and well-being of populations.

By recognizing the importance of resilient skills for the efficient performance of health systems, these concepts bring the framework proposed by the WHO closer to the Resilience Engineering^16,17^ approach, according to which complex systems increase their capacity to adapt and recover from shocks as they improve their abilities to monitor short-term risks and anticipate long-term threats, to learn from experience and thus to respond quickly and appropriately^18^ to unexpected events.

A more comprehensive notion of what resilient health systems should look like broadens the perspectives for research, management, and planning of public policies based exclusively on scientific evidence. With the focus on improving the functioning of the SUS's organizations and services, the importance of incorporating resilience as an essential aspect that must be constantly monitored and evaluated becomes increasingly clear.

Despite the difficulty of quantifying something that involves subjective capacities of systems and people, there are some attempts to measure resilience, such as the *Systemic Potentials Management* (SPM)^13^ and the Coefficient of Resilience(CoReS)^19^, the latter with a specific focus on public health. These metrics help to introduce resilience into known health planning and management frameworks, allowing existing indicators to be used to monitor and evaluate the SUS's potential for resilient performance.

Resilient performance must be evaluated based on regular operation, but it makes little sense to measure this potential based on indicators of past events exclusively or, worse, of disasters that have already occurred. Thus, a resilience coefficient for a public health system, organization, or service must face the challenge of delimiting elements that represent not resilience itself, but the potential for resilient performance.

In 2023, the term "Health Systems Resilience" was submitted to the Health Sciences Descriptors catalog (DeCS), initially as an alternative descriptor for the term "Health Systems." In 2024, this term should be classified as an independent descriptor, defined as: "the adaptive capacity that health systems must develop and maintain to adequately meet the sudden increase in demand caused by extraordinary events that affect the health of the population, directly or indirectly, concomitantly with maintaining the operation, safety, quality and availability of services"^20^ (our translation).

The consolidation of this definition represents a significant advance towards a more adherent conceptualization, capable of driving a change in the dynamics of how health organizations operate—the continuous development of skills to deal with stresses on their operation caused not only by major health disasters, but by any type of extraordinary event.

It is by developing this daily resilience that a complex system like the SUS can continually become more resilient to large-scale events such as natural disasters, disease outbreaks, and epidemics. Preserving permanent spaces for building adaptive capacity will enable health workers to develop new ways of dealing with unpredictability in a giant, diverse and unequal country like Brazil.
