# Supplementary material for: The resilience of the Brazilian Unified Health System is not (only) in responding to disasters
Source: Rev Saude Publica. 2024 Jun 6;58:22. doi: 10.11606/s1518-8787.2024058005731 (PMC11196091; doi:10.11606/s1518-8787.2024058005731)
Supplement: Translation

# Translation: A resiliência do Sistema Único de Saúde não está (somente) nas respostas aos desastres

**Authors:** Alessandro Jatobá, Paulo Victor Rodrigues de Carvalho

**Affiliations:** I Rio de Janeiro RJ Brasil Fundação Oswaldo Cruz. Centro de Estudos Estratégicos Antônio Ivo de Carvalho. Rio de Janeiro, RJ, Brasil

**Keywords:** Políticas de Saúde, Sistemas de Saúde, Gestão em Saúde

## Abstract

O enfrentamento da recente pandemia da covid-19 mostrou que o Sistema Único de Saúde (SUS) precisa aprimorar sua resiliência para lidar com o rápido alastramento de enfermidades transmissíveis sem deixar de garantir o cuidado necessário a uma população envelhecida, com comorbidades e em situação vulnerável. Este artigo identifica, analisa e discute aspectos críticos da resiliência do SUS, colocando em xeque o foco prevalente sobre a robustez e o volume dos recursos mobilizados emergencialmente na deflagração de grandes desastres. Recentes estudos demonstram que as habilidades que favorecem a adaptação a situações inesperadas emergem a partir do funcionamento cotidiano das organizações. Restringir a discussão ao simples dimensionamento das estruturas para reagir aos eventos adversos tem o efeito de engessar o seu potencial, inibindo o afloramento de habilidades transformadoras, adaptativas, de antecipação e de aprendizado necessárias para o desenvolvimento sustentável da resiliência.

## INTRODUÇÃO

Normalmente, quando se fala sobre o assunto da resiliência das organizações de saúde, imediatamente pensamos no arsenal de componentes emergenciais que essas instituições mantêm para implementar as fases do ciclo de resposta aos desastres sanitários, envolvendo preparação, mitigação, resposta e recuperação^1^. Um dos motivos para essa associação é o fato de que, durante muito tempo, as organizações de saúde acreditavam que, para fortalecer sua resiliência, bastaria manter ou desenvolver os recursos físicos, financeiros e estruturais adequados para enfrentar o surgimento de intercorrências emergenciais. Afinal, de acordo com as definições correntes, os desastres sanitários são eventos intensos, inesperados, repentinos e incomuns^2^.

De fato, essa noção de resiliência com foco na resposta a desastres é indispensável. No entanto, como a amarga experiência ocasionada pela pandemia da covid-19 se encarregou de demonstrar, esses cuidados são insuficientes. Isso ficou evidente quando os sistemas de saúde dos países mais ricos, considerados sólidos e seguros, foram fortemente impactados pela rápida disseminação e capacidade destrutiva do vírus SARS-CoV-2^3,4^.

Foi preciso um massacre sanitário global para se estabelecer uma nova convicção – ter estruturas robustas é necessário, mas não é suficiente para garantir o desenvolvimento da resiliência de uma organização de âmbito nacional como o nosso Sistema Único de Saúde (SUS). Além dos instrumentos para lidar com os desastres, a literatura especializada mais recente aponta para a importância do desempenho contínuo no cotidiano dos sistemas de saúde, em situações de normalidade.

Haldane e Morgan^5^ argumentam que as duras lições aprendidas durante a pandemia representam uma oportunidade para superar problemas e desigualdades estruturais antigas no setor da saúde, de forma a otimizar o funcionamento dos sistemas e tornar o ambiente mais sustentável. Haldane et al.^6^ examinaram práticas na Atenção Primária à Saúde (APS) de diversos países, inclusive o Brasil, indicando que a resiliência no enfrentamento da covid-19 dependeu fortemente do quanto tais práticas estavam disseminadas e desenvolvidas, para além das estruturas dos serviços e do volume de recursos físicos e financeiros disponíveis. Arcuri et al.^7^ evidenciaram as possibilidades de adaptação das equipes do Serviço de Atendimento Móvel de Urgência (Samu-192) na região do Alto-Solimões (AM) perante o aumento da demanda e dos riscos no acolhimento aos usuários durante a covid-19, antecipando capacidades adaptativas e fragilidades a partir do funcionamento do serviço de embarcações-ambulância (popularmente chamadas de "ambulanchas"), para a garantia do acesso das populações ribeirinhas ao atendimento de saúde.

No que se refere à governança dos sistemas, a literatura recente demonstra como o funcionamento inadequado dos arranjos organizacionais comprometeu a resiliência durante a pandemia, mesmo com a mobilização de estruturas robustas e recursos extraordinários. Nesse sentido, Neves et al.^8^ exploraram as articulações entre as partes interessadas e os órgãos envolvidos no enfrentamento da pandemia no Brasil, indicando que a incoerência de discurso e desarticulação nas comunicações oficiais interferiu negativamente na mobilização dos esforços para frear o avanço da doença. Carvalho et al.^9^ mostram que a agenda política em diferentes níveis e setores do governo prejudicou as capacidades do sistema para monitorar, responder, antecipar e aprender, aspectos essenciais do desempenho resiliente. Chioro et al.^10^ vão mais além, afirmando que a resiliência do SUS depende fundamentalmente de regimes democráticos, em que os princípios básicos da universalidade, integralidade e equidade sejam respeitados.

O recente reconhecimento pelas autoridades sanitárias da importância da resiliência como um atributo fundamental a ser desenvolvido e gerido nos sistemas nacionais de saúde é um sinal positivo de progresso. Entretanto, ainda precisamos avançar em direção a uma conceituação que considere amplamente o funcionamento dos sistemas de saúde e a articulação entre os níveis de atenção em favor do desenvolvimento contínuo de capacidades preventivas, absortivas e adaptativas, incorporando, assim, possibilidades adicionais de lidar com os diferentes tipos de ameaças às quais as organizações de saúde pública estão cotidianamente sujeitas.

## UMA CONCEITUAÇÃO ADERENTE AO SUS

Primeiramente, é preciso destacar que, de fato, a resiliência dos sistemas complexos envolve conseguir responder a choques ou desastres. No entanto, no caso da saúde pública, a noção de choque não se limita a um evento disruptivo de grande porte. Eventos inesperados ocorrem o tempo todo, e mesmo ocorrências de menor intensidade podem ter forte potencial destrutivo. As práticas amplamente conhecidas e disseminadas estão sujeitas à repentina necessidade de adaptações, dada a complexidade do provimento de serviços de maneira integral e universal, especialmente na sociedade diversificada de um país desigual como o Brasil.

A resiliência dos sistemas públicos e universais de saúde é dependente da forma como suas organizações se articulam para funcionar e se comportam diante das adversidades cotidianamente enfrentadas pelas populações que devem assistir. Por isso, as capacidades que garantem a segurança e a robustez dos sistemas devem ser geridas adequadamente em favor da resiliência, incluindo a operação ininterrupta, resolutiva e de qualidade de um conjunto mínimo de funções essenciais^11,12^. Provavelmente, esse foi um dos problemas que prejudicaram a resposta à pandemia em países como os Estados Unidos e o Brasil. Embora dispusessem de estruturas robustas, tiveram de lidar com dificuldades de operação e articulação nos diversos níveis dos seus sistemas de saúde.

No caso brasileiro, ocorreram problemas importantes, como as estratégias de comunicação e monitoramento, a adoção de medidas não farmacológicas, a aquisição de vacinas etc. Essas questões extrapolaram a mobilização de recursos físicos e financeiros para o SUS, relacionando-se diretamente com a forma de operação do sistema durante a pandemia, desde seus arranjos de governança até o cuidado na ponta. Com o enfraquecimento das medidas de distanciamento social, o saturamento na capacidade de atendimento levou à suspensão de cirurgias eletivas e tratamentos para doenças crônicas em alguns locais, por exemplo.

Embora tenham demonstrado potencial para resiliência ao disponibilizar recursos para enfrentar algumas consequências da pandemia, o SUS e outros sistemas considerados seguros e robustos apresentaram fragilidades que não podem ser desprezadas.

Segundo Hollnagel^13^, não se trata de um sistema *ser* resiliente ou *ter* resiliência, mas sim *funcionar* de forma mais ou menos resiliente, o que envolve ter sucesso sob condições variáveis. Mais precisamente, o desempenho resiliente pode ser entendido como uma condição dinâmica e contínua, na qual os problemas estão momentaneamente sob controle devido a mudanças compensatórias providenciadas pelos atores envolvidos. Isso é essencial para o SUS, que tem a missão de fornecer acesso universal, equânime e cuidado integral em um contexto no qual mudanças inesperadas podem surgir a qualquer momento, com consequências capazes de se propagar rapidamente.

As autoridades sanitárias devem desenvolver uma cultura de transformação permanente que propicie o aprimoramento contínuo de habilidades preventivas, absortivas e adaptativas, de modo a permitir que tanto as organizações quanto as pessoas aprendam com a experiência, monitorem e antecipem riscos e, assim, respondam adequadamente, tanto aos acontecimentos menores quanto aos grandes eventos de saúde pública. É preciso mobilizar e articular suas estruturas, para que o desempenho resiliente sempre possa emergir diante de um evento inesperado, independentemente de sua intensidade ou potencial destrutivo.

## CENÁRIOS E PERSPECTIVAS PARA PESQUISA E POLÍTICA PÚBLICA

Como parte dos esforços para enfrentar as consequências das mudanças climáticas, a Organização Mundial da Saúde (OMS) propôs no bojo dos Objetivos de Desenvolvimento Sustentável (ODS) da Agenda 2030 um arcabouço conceitual para promover o desenvolvimento de capacidades adaptativas dos sistemas de saúde^14,15^. Para a OMS, a resiliência pode ser fortalecida a partir de um conjunto de habilidades, como percepção, transformação, mobilização, autorregulação, integração e diversidade, todas elas focadas na sustentação de um nível mínimo de operação contínua de funções essenciais para a saúde e o bem-estar das populações.

Ao reconhecer a importância das habilidades resilientes para a eficiência do desempenho dos sistemas de saúde, esses conceitos aproximam o arcabouço proposto pela OMS da abordagem da Engenharia de Resiliência^16,17^, segundo a qual os sistemas complexos aumentam sua capacidade de se adaptar e se recuperar de choques à medida que aprimoram suas habilidades de monitorar riscos de curto prazo e se antecipar a ameaças de longo prazo, aprender com a experiência e, assim, responder rápida e adequadamente^18^ a eventos inesperados.

Uma noção mais abrangente de como devem ser os sistemas de saúde resilientes amplia as perspectivas para a pesquisa, gestão e o planejamento de políticas públicas baseadas exclusivamente em evidências científicas. Com o foco assentado sobre a melhoria do funcionamento das organizações e serviços do SUS, fica cada vez mais clara a importância de se incorporar a resiliência como um aspecto essencial, que deve ser constantemente monitorado e avaliado.

Apesar da dificuldade de quantificar algo que envolve capacidades subjetivas dos sistemas e pessoas, existem algumas tentativas de mensuração da resiliência, como o *Systemic Potentials Management* (SPM)^13^ e o Coeficiente de Resiliência em Saúde (CoReS)^19^, este último com foco específico em saúde pública. Essas métricas ajudam a introduzir a resiliência nos arcabouços conhecidos de planejamento e gestão em saúde, permitindo o aproveitamento de indicadores já existentes para monitorar e avaliar o potencial do SUS para o desempenho resiliente.

É preciso considerar o desempenho resiliente a partir do funcionamento regular, mas faz pouco sentido medir esse potencial a partir de indicadores que apontam apenas para eventos passados ou, pior que isso, para desastres já ocorridos. Assim, o coeficiente de resiliência de um sistema, organização ou serviço público de saúde deve enfrentar o desafio de delimitar elementos que representam não a resiliência em si, mas o potencial para o desempenho resiliente.

Em 2023, o termo "Resiliência de Sistemas de Saúde" foi submetido ao catálogo de Descritores em Ciências da Saúde (DeCS), inicialmente como descritor alternativo do termo "Sistemas de Saúde". Em 2024, esse termo deve ser classificado como um descritor independente, definido como: "a capacidade de adaptação que os sistemas de saúde devem desenvolver e manter para atender de forma adequada ao repentino aumento da demanda causada por eventos extraordinários que afetam a saúde da população, direta ou indiretamente, concomitante à manutenção do funcionamento, segurança, qualidade e disponibilidade de serviços"^20^.

A consolidação dessa definição representa um avanço significativo em direção a uma conceituação mais aderente, capaz de impulsionar uma mudança na dinâmica de funcionamento das organizações de saúde – o desenvolvimento contínuo de habilidades para lidar com tensões sobre sua operação causado não apenas por grandes desastres sanitários, mas por qualquer tipo de evento extraordinário.

É com o desenvolvimento dessa resiliência cotidiana que um sistema complexo como o SUS pode se tornar continuamente mais resiliente a ocorrências de grande porte, como desastres naturais, surtos de doenças e epidemias. A preservação de espaços permanentes para a construção da capacidade adaptativa dará aos trabalhadores da saúde condições para desenvolver novas formas de lidar com a imprevisibilidade em um país gigante, diversificado e díspar como o Brasil.
